# Healthcare-Associated Invasive Mould Diseases: From Rapid Diagnostics to Safe and Effective Management

**DOI:** 10.3390/jof12100710

**Published:** 2026-09-22

**Authors:** Sebastien Imbert, Annalise Unsworth, Priya Garg, Justin Beardsley, Catriona L. Halliday, Sharon C.-A. Chen

**Affiliations:** 1 Centre de Recherche Cardio-Thoracique de Bordeaux, INSERM U1045, Université de Bordeaux, FR-33318 Pessac, France; 2 Sydney Infectious Diseases Institute, Faculty of Medicine & Health, The University of Sydney, Sydney, NSW 2145, Australia; 3 Centre for Infectious Diseases and Microbiology—Public Health, Westmead Hospital, Sydney, NSW 2145, Australia; 4 Institute of Clinical Pathology and Medical Research, New South Wales Health Pathology, Sydney, NSW 2145, Australia; 5 Department of Infectious Diseases, Westmead Hospital, Sydney, NSW 2145, Australia; 6 National Centre for Infections in Cancer, Peter MacCallum Cancer Centre, Melbourne, VIC 3000, Australia; 7 Faculty of Medicine, Dentistry and Health Sciences, University of Melbourne, Melbourne, VIC 3053, Australia; 8 Centre for Infectious Diseases and Microbiology—Laboratory Services, Westmead Hospital, Sydney, NSW 2145, Australia

**Keywords:** invasive fungal infection, healthcare-associated infection, infection control, genotyping

## Abstract

Invasive mould diseases (IMDs) are an important component of healthcare-associated infections (HAIs). However, they can be difficult to identify, as the standard definition of HAIs do not always apply and usually have a longer and more variable onset after exposure. Identifying the source of infection is also challenging, as moulds can colonise a wide range of indoor healthcare environments, and multiple sources of exposure are possible. Thus, healthcare-associated IMDs may reflect a broader failure of infection control measures rather than a single isolated event. In addition, IMDs may evolve into outbreaks that are hard to detect and investigate, as they can develop over extended periods before raising concern. Here, we review mould ecology in healthcare settings and describe the patient groups at risk for healthcare-associated IMDs. We also overview the investigative approaches used to identify case clusters—including case finding, environmental sampling and isolate genotyping—summarise major IMD outbreaks and highlight best management practices. Taken together, this knowledge will help identify high-risk situations and guide the implementation of targeted preventive measures and diagnostic strategies that enable the timely diagnosis and effective management of healthcare-associated IMDs.

## 1. Introduction

Healthcare associated-infections (HAIs) are infections that are acquired directly or indirectly through contact with a healthcare setting [1,2]. They include infections acquired in hospital or in other healthcare environments, as well as those following medical or surgical procedures. As such, HAIs are typically defined as infections occurring ≥48 h after admission or within 30 days after discharge, and they are notable for their substantive morbidity, mortality, and excess healthcare costs [2,3]. HAIs frequently involve antimicrobial-resistant pathogens, including multi-drug-resistant ones. A substantial proportion of HAIs can be avoided through effective infection prevention and control measures.

Invasive mould diseases (IMDs) are an important component of HAIs because they mainly affect immunocompromised or critically ill patients who require prolonged stays in healthcare facilities and have attendant adverse outcomes [4,5]. However, the standard definition of HAIs may not apply to IMDs given the multiple possible sources of exposure and the longer and more variable onset after exposure [6]. In addition, the sources of IMDs are often difficult to pinpoint, with predominantly exogenous exposure being responsible, whereby moulds are ubiquitous and colonise a broad range of indoor healthcare environments or medical devices [7,8,9]. Beyond individual patient cases, IMDs may also evolve into potential clusters and outbreaks that may be difficult to detect and investigate, as they can evolve over extended periods before raising concern [10]. Conversely, moulds can also contaminate sampling devices or laboratory materials, leading to pseudo-outbreaks that must be distinguished from true outbreaks to avoid unnecessary interventions [11].

In this review, we will firstly focus on the ecology of mould in healthcare settings to better understand potential sources of exposure, then describe the patient groups at risk for healthcare-associated IMDs. We also review major IMD outbreaks and describe the case-finding approaches, diagnostic tools used to investigate case clusters, and management strategies.

## 2. Mould Ecology in Healthcare Settings

Moulds are saprophytic fungi that are found and accumulate in multiple environments, including indoor healthcare settings. A good understanding of their ecology is essential to assess the risk of healthcare-associated IMDs and to adopt prevention strategies.

Moulds can disseminate from outdoor sources into indoor healthcare environments by producing airborne conidia, which can remain suspended in air for hours [12]. Indoor conidia concentrations in non-protected areas generally reflect those of the outdoor environment, where the concentration can exceed 1000 conidia/m^3^, with the low-pathogenic genera *Cladosporium* and *Alternaria* being the most abundant [13]. Of note, the highly pathogenic *Aspergillus* genus is typically present at lower concentrations (0.2 to 15 conidia/m^3)^, although levels can exceed 100 conidia/m^3^ under specific conditions [14]. Qualitative and quantitative variations in conidia loads have been documented, with the highest concentrations typically observed in summer and with seasonal shifts in the dominant genera [13,15]. Proximity to construction work or to composting sites can further increase conidia loads.

Moulds can also disseminate in healthcare settings from indoor sources, particularly through ventilation ducts where conidia can accumulate [16]. The regular cleaning of ventilation systems is therefore necessary to prevent contamination of the controlled areas [16]. However, cleaning procedures themselves can transiently increase airborne spore concentrations [16]. Water can also serve as a source of dissemination and contamination in healthcare settings, but it is not known if transmission occurs directly from water or following aerosolization [17,18,19]. In addition, water damage and leaks can favour local mould proliferation. Dusty areas represent another mould hotspot within indoor healthcare environments, as moulds can accumulate in settled dust, necessitating regular cleaning in patient care zones. Storage rooms likewise require careful management to prevent the contamination of materials or medical devices, including incubators, linen, needles or adhesive bandages [7,8,9,20,21].

As moulds are also major phytopathogens, they can be found in potted plants and fresh flowers, as well as on raw fruits and vegetables. Other food products that have been implicated as potential sources of mould infection in immunocompromised patients include outdoor-dried items such as tea, pepper, or other spices and milk-derived products such as cheese or yogurt [8,22,23,24].

Finally, although the patient-to-patient transmission of IMDs is very uncommon, infected patients themselves have been reported as sources of contamination for others: for instance, in the context of mould-infected wounds [25,26], or patients with high pulmonary *Aspergillus* burdens [27]. Conversely, no transmissions of IMDs through healthcare workers have been reported to date.

## 3. Definitions and Challenges in Healthcare-Associated IMDs

The defining and detection of healthcare-associated IMDs are challenging, given (i) the heterogeneity of at-risk patients, (ii) the spectrum of pathogens and their differencing ecology, and (iii) the lag between exposure and infection [6]. Hence, the time and place of acquisition is often uncertain unless there is a clear source, e.g., contaminated injectable product. Airborne exposure to the pathogen usually results in a period of colonization prior to infection, yet early clinical and radiological abnormalities are frequently absent, complicating timely diagnosis. In addition, IMDs are usually diagnosed in hospital settings in patients who frequently attend healthcare facilities, making it difficult to distinguish community-acquired secondary IMDs in at-risk individuals from true healthcare-associated IMDs.

Meijer et al. proposed definitions for nosocomial aspergillosis in immunocompromised patients, classifying cases as “possible” when they occur between 7 and 14 days after hospital admission and as “probable” when they manifest more than 14 days after admission or in patients who were discharged within the preceding 14 days [6]. However, these definitions are unlikely to apply uniformly across all patient populations or mould pathogens. Studies have shown that the observed incubation period may range from a couple of days—as short as 48 h in trauma- or burn-related IMD cases—to weeks in haematological malignancy or many months post-organ transplant [28,29].

Grouped cases or outbreaks of healthcare-associated IMDs may be difficult to identify due to their relatively low incidence and the absence of consensus definition. In its simplest form, an outbreak is defined as an increase in the usual incidence of infection [8]; however, this requires knowledge of the baseline incidence. Another proposed definition considers an outbreak to be present when ≥2 cases of a particular IMD are linked in time and place. Finally, outbreaks have also been reported for IMDs involving different mould species or genera (e.g., for mucormycosis).

Grouped cases and outbreaks can reflect an issue in the prevention procedure and/or an unanticipated event. In both situations, they must be investigated to prevent further cases. Atypical IMDs (i.e., IMDs presenting with unusual symptoms, occurring in non-classical populations or involving uncommon pathogens relative to the local epidemiology) should likewise be investigated, as they are more likely to be healthcare-associated. This approach requires robust knowledge of the local epidemiology and therefore systematic surveillance of IMDs diagnosed within a healthcare institution. A conceptual framework for initiating healthcare-associated investigations is presented in Figure 1.

## 4. Patients at Risk of Healthcare-Associated IMDs

A broad range of patients are at risk of healthcare-associated IMDs, from those considered high-risk, who require strict preventive measures and usually present with characteristic radio-clinical features, to lower risk patients in whom IMDs are less predictable than high-risk ones and clinical presentations are often atypical.

### 4.1. High-Risk Patients

#### 4.1.1. Haematology Patients

Patients with haematological malignancy and those undergoing haematopoietic stem-cell transplantation (HSCT) are considered to be at a high risk of IMD, with the greatest vulnerability observed in the acute leukaemia and allogeneic HSCT cohorts [30,31]. Key predisposing factors include prolonged neutropenia (whether in the context of disease or treatment-related factors), relapsed or refractory disease, severe graft versus host disease (GvHD) and long-term corticosteroid use [30]. Traditional assessments of fungal risk have been further complicated by the expanding use of novel disease-related immunosuppressive therapies, with increased IMD susceptibility linked to a broad range of targeted treatments including anti-CD52, TNF-α, Janus kinase (JAK), BTK and PI3K inhibitors [31]. Evidence in chimeric antigen receptor (CAR) T-cell-treated sub-groups is also emerging and, though rare, thought to be greatest in those with previous IMDs, prolonged cytopenia and or cytokine-release syndrome/immune-effector cell-associated neurotoxicity syndrome (CRS/ICANS), particularly where further immunomodulation may be required [32]. Similarly, data on IMDs following bispecific antibody (BsAb) therapy, primarily used in multiple myeloma, remains limited, and surveillance is recommended [33,34].

Clinical presentation varies with the pathogen, with pulmonary, sinus and disseminated disease the most typically reported anatomical sites [35]. Thus, an increased degree of suspicion for IMDs is recommended among high-risk patients presenting with new respiratory symptoms and persistent fever of uncertain aetiology [35]. Beyond antifungal prophylaxis, control mechanisms within the healthcare setting include strict environmental precautions such as the use of high-efficiency particulate air (HEPA) filtration to maintain protected air quality and positive-pressure ventilation (PPV) in high-risk areas [36]. Periodic airborne fungal sampling may also be of benefit in areas where opportunistic fungi are of concern (see later) [36,37,38]. Data on the efficacy of a low microbial diet on reducing IMDs are sparse, though previous studies have supported recommendations to avoid all foods where prior sterilisation or disinfection is not implemented [24,39].

#### 4.1.2. Lung Transplant Recipients

Lung transplant recipients experience the highest burden of IMDs among solid organ transplant (SOT) groups and, from the perspective of environmental mould exposure and outbreak prevention, are appropriately considered alongside other high-risk populations such as patients with acute leukaemia and allogeneic HSCT [40,41,42].

Incidence rates of ~4–9% for IMDs in the year following transplant are frequently described [43,44], but one cohort of 274 patient recently reported an invasive aspergillosis (IA) incidence of 32%, where the median duration to the onset of disease was 6 months post-transplant [45]. Lung transplant recipients are especially susceptible to IMDs through a combination of direct environmental exposure of the allograft to inhaled fungal spores, impaired mucociliary clearance, reduced cough reflex, structural abnormalities, and long-term significant immunosuppression. Additional recognised risk factors include pre- and post-transplant mould colonisation, cytomegalovirus (CMV) infection, airway stenosis, chronic lung allograft dysfunction, renal replacement therapy and episodes of rejection requiring intensified immunosuppression [45,46,47]—with such patients calling for extra vigilance.

#### 4.1.3. Burn Patients

Patients with major burns represent a distinct risk group for IMDs due to the extensive disruption of the skin barrier, prolonged ICU admission, broad-spectrum antibiotic exposure, repeated surgical procedures and burn-associated immune dysfunction [48]. In one large contemporary series, over 20% of burn patients involving >15% of body surface area developed an IMD [48]. Infection frequently results from the direct inoculation of damaged tissue rather than inhalational acquisition. *Aspergillus* species and Mucorales are the most frequently reported pathogens, but *Fusarium* and other environmental moulds may be increasing in frequency [49,50]. Infections in burn units have been linked to environmental contamination, contaminated dressings, and construction-associated spore dispersal [50].

Clinical manifestations range from invasive wound infection and soft tissue necrosis to disseminated disease [48,50,51]—these infections are serious, with rapid progression and high mortality. Early diagnosis is challenging because fungal wound infection may be clinically indistinguishable from bacterial infection, colonisation, or non-infectious tissue necrosis. A high index of suspicion combined with early tissue sampling and screening for fungal infection is therefore essential to obtain the best outcome for patients.

### 4.2. Intermediate Risk Patients

#### 4.2.1. Other Organ Transplant Recipients

Although mould infections are less frequent in other SOT populations, they remain an important cause of morbidity and mortality. Liver transplant recipients experience the next highest burden of IMDs [40,42], with most infections occurring within the first few months after transplantation and often in the context of surgical complications, reoperation, renal replacement therapy, CMV infection or prolonged healthcare exposure [46,52,53]. Heart transplant recipients are similarly vulnerable during the early post-transplant period, particularly those requiring prolonged intensive care support, delayed chest closure, mechanical circulatory support or T-cell depleting therapies [54,55]. Notably, healthcare-associated aspergillosis outbreaks have been described in cardiothoracic and heart transplant units, emphasising the importance of environmental controls in these settings [56]. In contrast, kidney transplant recipients generally experience a lower incidence of IMDs, with most cases occurring beyond the first post-transplant year and in association with intensified immunosuppression or treatment for rejection [40,42,57].

#### 4.2.2. Intensive Care Patients

IMDs are increasingly recognised in non-neutropenic intensive care units (ICUs). The pooled prevalence rate for the most common mould—*Aspergillus*—among ICU patients was 10% in a recent meta-analysis [58]. Important predisposing factors include prolonged mechanical ventilation, chronic lung disease, corticosteroid exposure, broad-spectrum antimicrobial use, extracorporeal membrane oxygenation (ECMO) and prolonged ICU admission [58]. Particular attention has focused on patients with severe viral pneumonitis, in whom influenza-associated pulmonary aspergillosis (IAPA) and COVID-19-associated pulmonary aspergillosis (CAPA) have emerged as important causes of morbidity and mortality [59]. Airway epithelial damage, immune dysregulation and corticosteroid therapy are thought to act synergistically to increase susceptibility to IMDs in these patients. Healthcare-associated exposures may contribute to additional risk through construction activities, ventilation failures, water damage and contaminated environmental reservoirs, particularly in units housing prolonged mechanically ventilated patients, which therefore necessitate specific prevention measures.

Unlike patients with haematological malignancy, radiological findings are often non-specific, and diagnosis is challenging [59]. A high index of suspicion combined with early bronchoscopy and bronchoalveolar lavage screening for fungal infection is therefore essential to optimise patient outcomes.

### 4.3. Low or Background Risk

Most patients visiting healthcare settings have a low or background risk of IMDs. Although certain situations, such as the presence of internal or external devices [60], deep wounds [26], or premature neonatal status [20,61] may increase this risk, these conditions alone are not sufficient to develop IMDs. In these populations, the development of IMDs will likely reflect healthcare-associated factors, such as exposure from the environment or devices (e.g., incubators in premature neonates) or direct inoculation through contaminated material or devices, and therefore warrant investigation. Transplant tourism [62] and cosmetic surgery for aesthetic reasons [63] have likewise been associated with healthcare-associated IMDs. In addition, IMDs have also been documented in patients without specific risk factors following the injection of a contaminated product [64,65,66,67].

Although infection is usually localised to the site of inoculation, the clinical presentation in these low-risk patients may be atypical and can involve uncommon or more unusual fungi, for which establishing accountability for causality may be challenging, thereby delaying diagnosis.

## 5. Investigation of Healthcare-Associated IMDs

The main elements of investigation include case finding, environmental assessment with the consideration of targeted environmental sampling, and comparative mycological and genotypic analyses interpreted in conjunction with temporal, spatial and epidemiological information.

### 5.1. Diagnosis of IMD and Case Finding

The diagnosis of IMDs involves an assessment of host risk factors, clinical features and mycological findings. It can include targeted or screening strategies regarding whether the patient is symptomatic or not. In either case, mycological findings should be interpreted cautiously, considering the known diagnostic performances and the potential risk of environmental contamination. The main diagnostic elements are summarised in Table 1.

#### 5.1.1. Targeted Diagnosis

In symptomatic patients, investigation includes radiological imaging to assess the site and extent of infection and the biopsy of the involved sites. In identifying an index case, definitive diagnosis relies on the isolation of mould from biopsies, sterile sites, body fluids and blood cultures, as well as direct microscopy and histopathology. Methods and techniques of culture and microscopy-based diagnosis have been well described in consensus definitions and diagnostic guidelines [68,69,70,71,72]. Briefly, a proven case requires the isolation or the amplification and DNA sequencing of a mould from a sterile site or blood culture, or histopathological, cytopathologic or direct microscopic examination of a mould with associated tissue damage [68]. In addition to host factors and clinical features, the mycological evidence of a probable case includes a positive culture from a non-sterile site (such as sputum, bronchial alveolar lavage fluid, or wound biopsy), as well as the detection of circulating fungal antigens and nucleic acids [68]. During the investigation of a potential cluster, the diagnosis of patients with clinical symptoms follows a similar approach to that of the index case.

#### 5.1.2. Screening

In addition to targeted diagnosis in symptomatic patients, screening for IMDs may also be useful to improve early detection in high-risk asymptomatic patients or during investigations of potential clusters. Screening protocols for *Aspergillus* infections have been established in haematology patients with prolonged neutropenia, and serum biomarker screening may also be considered in burn patients [51,73,74]. Elevated serum galactomannan levels, as well as positive serum *Aspergillus* PCR results, can precede the first clinical or radiological signs of infection by several days, enabling prospective monitoring [75,76].

Screening approaches for other moulds have been described in high-risk patients, although they have not yet been incorporated into clinical guidelines. Notably, a positive Mucorales PCR in serum can appear 4–9 days before diagnosis and 1–2 days before imaging [77,78,79], and it has been used in both burn and haematology patients to initiate earlier treatment [80,81]. Similarly, serum *Fusarium* PCR has been shown to detect infection up to one week before confirmation by blood culture or biopsy [82]. Microbial cell-free DNA (mcfDNA) metagenomic testing in serum has been shown to detect pathogen DNA (including *Aspergillus* and Mucorales) up to three weeks prior to clinical diagnosis and may represent a promising screening tool [83]. Finally, in addition to serum screening, iterative bronchial alveolar lavage in lung transplant patients and serial wound sampling in patients with severe burns may also be used as IMD screening approaches in these high-risk populations [51,84].

### 5.2. Environmental Investigation and Sampling

The environmental sampling of hospital environments refers to the process of collecting microbiological samples from water, hard surfaces and air for pathogen detection, quantification and monitoring. For hard surface sampling, similar methods of sample collection used for clinical specimens, e.g., the use of swabs, may be performed, whereas for air sampling, either active air capture or the use of settle plates may be carried out [85]. Regardless of methodology, it can be difficult to distinguish between clinically important fungal isolates and contaminants, and the reporting of results requires care [86]. In addition, the interpretation of results is challenging due to the absence of universally accepted fungal burden thresholds to indicate whether the sampled area is “safe” for clinical use.

We first briefly describe common methods of sampling and then discuss the role of performing environmental investigations.

#### 5.2.1. Air Sampling

Active air sampling uses specialised equipment to draw a defined volume of air across an agar surface. Plates are incubated typically at 25–30 °C from 2 to 7 days, with results expressed as colony-forming units per cubic metre (CFU/m^3^), providing a quantitative and broadly representative assessment of airborne fungi [28,85]. A limitation of this approach is that the sampling process can disrupt airflow, potentially introducing bias into the measurements obtained [86].

Settle plates are a passive air sampling method in which viable fungal spores settle by gravity onto an agar surface over a defined exposure period, followed by incubation. Results are expressed as CFU per unit area per exposure time (CFU/m^2^ or cm^2^/time) but are not considered quantitative or qualitative [28,86]. Although simple and cost effective, settle plates are not recommended for fungal air monitoring, as they selectively collect larger particles while single spores can remain suspended in air indefinitely [87].

#### 5.2.2. Surface and Water Sampling

Surface sampling targets areas where fungal contamination is more likely, such as high-touch surfaces and equipment; however, its utility is limited by the lack of standardised methodologies and absence of established threshold values to establish unacceptable fungal burdens [88]. Although simple and inexpensive, the accuracy of surface sampling depends on appropriate sampling and analytical techniques, and it does not directly detect airborne fungi present at the time of sampling [28,85]. Combining surface and air sampling thus provides a more comprehensive assessment of fungi present than either surface or air sampling alone [85].

Water may also represent a potential source of fungi [89], but unlike bacteria, viruses and parasites, there are no standardised methods for assessing fungal contamination in water. The routine testing of hospital water is generally not indicated outside of outbreak investigations, and even then, only after careful consideration. Thresholds for nosocomial fungal contamination of water have not been defined [19,87,90].

#### 5.2.3. Role of Environmental Sampling in Healthcare-Associated IMDs

Environmental sampling may be undertaken routinely or performed in response to an event such as suspected case clusters of infection or a suspected or known compromise in building or environmental infrastructure, e.g., breaches in heating, ventilation and air conditioning (HVAC) systems or visible mould seen on otherwise clean surfaces. Table 2 summarises the authors’ considerations in a reasonable and pragmatic approach to performing environmental sampling in these clinical contexts.

##### Routine Sampling and Surveillance

Currently, there is no requirement for routine facility-wide environmental fungal surveillance in healthcare facilities [85] because there is lack of robust evidence to support this practice (Table 2) and, importantly, little to predict individual patient risk. However, despite evidence being limited, some authorities regard it as an important adjunct to other environmental precautions in high-risk patient areas, such as HSCT wards and ICUs [19]; these recommendations are largely based on expert opinion and driven by past experiences of the institution with mould outbreaks, and they should be assessed on a case-by-case basis. It is also important to recognize that there are differences between countries in building maintenance requirements that involve the measurement of air quality. If undertaken, this can assist in establishing institutional baselines in high-risk clinical units and detecting deviations from those baselines.

##### Event-Triggered Sampling

The United States (US) Centre for Disease Control (CDC) and other international guidance documents have suggested that environmental sampling may be positioned as a targeted tool to: (i) support outbreak investigation, (ii) monitor the efficacy of cleaning, and (iii) validate infection control protocols in healthcare facilities, particularly for immunocompromised patients (summarized in Table 2) [6,28,87]. This is also the practice in some Australian Healthcare facilities (authors personal communication). It is also important to acknowledge that, whilst sampling may be performed in the scenarios above, parameters such as the number of sampling locations and the size and physical area of environments to be sampled are most often determined arbitrarily. Further, how often to repeat the sampling will depend on the nature and magnitude of the event and if remediation work has been undertaken.

##### Role of Air Sampling in Outbreak Investigations

The role of air sampling in outbreak investigations continues to be heavily debated, yet it would be fair to state that, in a potential outbreak, air sampling is often inconclusive and seldom informs clinical decisions. It is sometimes performed in search of environmental sources in conjunction with water and surface sampling (see above) and is seen to be a proactive support approach. Of note, the timing of sampling will influence the result. For instance, when performed close to the onset of an environmental or clinical signal prior to any intervention, sampling may document elevated counts of airborne mould. In contrast, more delayed sampling will likely reflect post-intervention measures and no longer reflect the relevant exposure environment [10,91].

Evidence supporting a correlation between airborne mould burden and IFD is, at best, mixed. In haematology units, many studies including longitudinal surveys have observed IMDs without correlation to measured airborne counts [92,93,94]. Conversely, others have noted temporal links between elevated *Aspergillus* counts in air and increased rates of IA [10,95]. Similar associations have been observed outside the outbreak scenario [96,97]. Recently, in one US study, the load of *Aspergillus* species in paediatric haematology/oncology units showed a strong association with the IMD rate (15.9-fold elevation for an increase of 1 CFU/m^3^ [95% CI 2.8–90.7, *p* = 0.002]). Here, targeted periodic air monitoring was performed on the premise that it might identify periods during which patients are at an increased risk for IMDs.

Additional limitations of air sampling are that there are no mould burden thresholds to predict the risk of IMDs or that can distinguish a “safe” from an “unsafe” environment. Therefore, the results are unable to direct meaningful clinical action. Indeed, for most patients, a safe lower level of exposure may not exist, as infections have been observed at very low airborne counts in outbreak settings [98]; a continuous trend of dose–response relationship rather than a single threshold measurement is likely to be potentially more useful. Further, differences in host susceptibility limit the generalisability of any threshold that is considered with infection risk reflective of cumulative and dynamic exposure rather than at a single time point. Interpretation is further complicated by methodological differences across institutions, units, and sampling locations which contribute to baseline variability and limit the meaningful contextual interpretation of results in the absence of longitudinal data [96,99]. Hence, any positive air sampling, or indeed positive sampling of the environment, should trigger discussion between the relevant clinical units, infection prevention practitioners and the hospital executive and building representatives in decisions regarding patient placement, cleaning and further surveillance.

Finally, air sampling may underestimate environmental contamination when reservoirs are primarily surface associated. In an *A. flavus* outbreak in Denmark, there was a substantially greater fungal burden from surface samples than paired air samples [100]. Taken together, air sampling can be considered a tool triggered by an event or incident that has a role in quality assurance and that can support environmental assessment and remediation, but it has limited ability to establish causation or assign patient risk [19].

### 5.3. Mycological and Genomic Investigations

The first level of the mycological investigation of healthcare-associated IMDs is the accurate identification of the pathogen. The definitive identification of fungal isolates is typically confirmed through matrix-assisted laser desorption/ionisation time-of-flight mass spectrometry (MALDI-TOF MS) or the sequencing of the internal transcribed spacer region (ITS), beta tubulin, calmodulin and/or translation elongation factor genes [101,102,103]. Panfungal PCR assays can be performed directly on clinical specimens through the amplification of the ITS1 and ITS2 regions, followed by melting curve analysis or amplicon sequencing [101,104,105,106]. In histopathology-positive, culture-negative isolates, panfungal PCR is an important adjunct for diagnosis, particularly in identifying Mucorales, which only grows in 15–25% of clinical samples [107,108,109]. Comparing the identification obtained from the patient specimen with that from the environmental sampling or from other patients may be sufficient to support the suspected source of infection or to identify grouped cases, particularly when the involved pathogen is uncommon. For more common pathogens, such as *Aspergillus* spp., antifungal susceptibility profiles may assist in identifying potential case clusters [110].

The second level of mycological investigation is the genotyping and comparison of genotypes across isolates of the same species. Several genotyping methods have been used, each offering different performance metrics and resolution depending on the objectives [111]. Molecular fingerprinting methods, such as restriction fragment length polymorphism (RFLP), random amplification of polymorphic DNA (RAPD), and amplified fragment length polymorphism (AFLP) analyses, were initially widely used for investigating healthcare-associated IMDs because they are simple to perform, do not require prior knowledge of genetic information [26,112,113,114,115,116], and can be applied to a broad range of pathogens. However, results may be difficult to interpret and often lack sufficient resolution at the strain level. In contrast, tandem repeat-based genotyping methods, such as short tandem repeat (STR), variable number tandem repeat (VNTR), and multi-locus VNTR analysis (MLVA), provide good strain-level resolution but require species-specific protocols, restricting their use to a limited number of moulds. STR genotyping has been widely used for investigating healthcare-associated aspergillosis, supported by a standardized protocol and primer set for *A. fumigatus* (STR*Af*) [117]. Multi-locus sequence typing (MLST) provides good intra-laboratory reproducibility but also requires species-specific protocols and dedicated sequence-type (ST) databases. In addition, its resolution at the strain level is generally lower than tandem repeat-based methods. MLST has therefore been used only infrequently for investigating healthcare-associated IMDs, with the exception of the genus *Fusarium*, for which curated databases are available [60,118,119]. Finally, whole-genome sequencing (WGS) approaches are increasingly used—notably, those based on single-nucleotide polymorphism (SNP) analysis. WGS provides high-resolution analysis; however, SNP number thresholds for strain delineation are not yet fully defined for mould pathogens, vary across species and may lead to an over- or underestimation of diversity in some instances [120]. Core-genome MLST (cgMLST) and whole-genome MLST (wgMLST) represent high-resolution extensions of standard MLST, but their use remains limited by the confined availability of ST databases.

When investigating healthcare-associated IMDs, the choice of the genotyping method depends on the availability of validated STR protocols or curated ST databases for the suspected species as well as resources for WGS analyses. Combining methods with different strain-level resolutions may also be useful to refine the assessment of isolate relatedness [120]. Nevertheless, mycological investigations and genotyping data cannot be used alone to confirm or rule out healthcare-associated IMDs or outbreaks or to establish the mechanism of transmission or the epidemiological causality, and they must be interpreted alongside temporal, spatial, environmental, and clinical epidemiological information.

## 6. Main Outbreaks of Healthcare-Associated IMDs

### 6.1. Aspergillus Healthcare-Associated Outbreaks

Several healthcare-associated aspergillosis outbreaks have previously been reported, most often in patients with haematological malignancy and typically occurring in the context of airborne conidia dispersion during construction work [98]. Although *A. fumigatus* and *A. flavus* are the most frequently involved species in this setting, mycological and/or genotypic investigations usually have retrieved multiple species or genotypes across patients rather than a single one [10,20,61,112,121]. This pattern reflects shortcomings in environmental control measures, with multiple sources of exposure rather than the expansion of a unique strain from a specific source. This is also highlighted by environmental sampling (see above), which usually reveals a high overall fungal load rather than a dominant single species or genotype [10]. A similar pattern has also been observed in outbreaks related to contaminated or non-functional air-handling units [121]. Therefore, in this context, differences in genotypes between clinical and environmental isolates should not be used to rule out a potential source of contamination.

In some cases, clinical outbreak isolates have been genotypically linked to environmental isolates. This has occurred notably when a specific source of contamination has been identified, such as premature neonates’ incubators, contaminated building materials, or another patient with extensive external lesions [20,26,61,100]. However, even when clinical isolates are linked to an environmental one, there are very few outbreaks in which the same genotype has been found across several patients, making the involvement of a single strain unlikely. Finally, some of these *Aspergillus* outbreaks have evolved more or less silently over months or even years [10,20,100], further complicating their detection and delaying their management. Table 3 summarises the major *Aspergillus* healthcare-associated outbreaks where genotyping was performed.

### 6.2. Mucorales Healthcare-Associated Outbreaks

Mucorales are the second most frequently reported moulds involved in healthcare-associated outbreaks next to *Aspergillus*. They have typically occurred in association with contaminated healthcare supplies or other environmental sources and are described in detail by Walther and colleagues [123]. Of 28 healthcare-associated outbreaks, common sources characterized included contact with contaminated medical devices (40.7% of cases), e.g., bandages, wooden tongue depressors, linen, shelving, and air (31.3%). The number of cases in a cluster has ranged from two to 12; diverse patient populations have been affected. The most common pathogen was *Rhizopus* species, but any Mucorales can cause outbreaks. Contaminated hospital linen, including laundry carts, appears to be a major source of Mucorales infections, presumably caused by suboptimal washing, drying and storage procedures, and they have caused both cutaneous and lung mucormycosis in Hong Kong [124] and the USA, affecting immunocompromised and transplant patients including children [125,126,127].

Where genotyping has been performed, one study demonstrated clonal relatedness between all four haematology patients with lung infection due to *Cunninghamella bertholletiae* [113], but no environmental isolates were available for study. More recent studies found that, albeit the number of isolates was relatively small, there was very high genetic diversity amongst case isolates (Table 4), with similar high diversity amongst non-outbreak and environmental isolates [114,120,128,129]. All studies showed that the clusters were due to multiple unrelated strains present in the environment, suggesting shortcomings in control measures as for most of the *Aspergillus* outbreaks.

### 6.3. Other Healthcare-Associated Mould Outbreaks

The knowledge of regional and institutional epidemiological patterns of this heterogeneous group of pathogens is key to diagnosis and management, yet outbreaks often have common themes of likely point sources: inadvertent exposure to contaminated products or devices including post-surgical events, exposure to contaminated environmental surfaces, or through the atmosphere. The numerous reports of healthcare-associated outbreaks caused by various pathogens are beyond the scope of the present review but can be found elsewhere [123,130,137]. We, however, summarise the major outbreaks where genotyping approaches have been used to support epidemiological clustering in Table 4. This was notably the case for *Scedosporium* and *Lomentospora* species, in one and two outbreaks, respectively, where genotyping identified identical genotypes across patients [115,116,132]. In addition, in one *Lomentospora* outbreak, clinical and environmental isolates shared the same genotype, corroborating the source of infection [116].

We also draw particular attention to clusters due to contaminated medicinal products and devices because of the cautionary lessons that must be learned. Historically, such healthcare-associated mould outbreaks focussed on contaminated contact lens solutions and post-cataract surgery contexts caused mainly by *Fusarium* species (summarised by Douglas et al. [130]). In one of the largest outbreaks, where 156 cases of *Fusarium oxysporum* species complex (FOSC) endophthalmitis occurred post-cataract surgery, identical genotypes were identified between clinical isolates and isolates cultured from ocular viscoelastic devices, implicating these devices as the source of infection [60]. Other post-cataract surgery outbreaks of *Fusarium* infection have occurred; in one Turkish study, nine consecutive patients of *Fusarium solani* species complex (FSSC) endophthalmitis had their procedure in the same operating theatre, but no environmental source was identified [138]. In addition, two concurrent outbreaks of endophthalmitis due to *Fusarium incarnatum-equiseti* species complex and *Bipolaris hawaiiensis* (now *Curvularia hawaiiensis*), respectively, were reported to have arisen from the use of contaminated products post-intraocular installation [133].

In 2012, the first outbreak of fungal meningitis of over 750 patients due to *Exserohilum rostratum* in the US was reported to have arisen from the administration of contaminated methylprednisolone through epidural, paraspinal, or joint injections, with mortality close to 8% [67], raising the level of public awareness. Genotyping revealed near-identical genomes among 18 isolates, suggesting a common source of infection [135]. Most recently, two outbreaks of FSSC meningitis were reported in Mexico and the US (mortality ≈80%) associated with epidural anesthesia received from May 2022 to February 2023 during surgical procedures in Durango and Matamoros, Mexico [64,65]. Although not all strains underwent genotyping, there was evidence to suggest this point source of infection (Table 4).

### 6.4. Pseudo-Outbreaks

Apart from true outbreaks, moulds can also be responsible for pseudo-outbreaks, which must be recognized to avoid unnecessary infection control measures and antifungal treatment. Pseudo-outbreaks may arise from laboratory contamination: for example, within incubators or biosafety cabinets. Such situations can be managed and investigated directly in the laboratory by re-analysing the suspected contaminated samples after judicious specimen tracking and appropriate cleaning procedures to exclude a true outbreak. However, sampling devices themselves can suffer from contamination. Under these circumstances, pseudo-outbreaks may be more difficult to detect, as re-analysis of the same sample will still yield a positive culture. Moreover, as the source of contamination is usually unique, isolate genotyping would identify the same genotype across samples and patients. Recently, a pseudo-outbreak affecting 22 patients with suspected *Purpureocillium lilacinum* cutaneous infection over a 10-month period was reported [11]. All skin samples had been collected by swab scraping in the same clinic. However, investigations revealed that the clinical context and skin lesions were not consistent with *P. lilacinum* infection. In addition, the refilled saline squeeze bottles used during swab sampling were contaminated with *P. lilacinum*. WGS SNP genotyping further demonstrated that the same genotype was shared between a clinical sample and the bottles, confirming the device source of contamination. This pseudo-outbreak highlights the importance of case finding and of assessing the accountability of a rare mould as a cause of healthcare-associated IMDs.

## 7. Management of Healthcare-Associated IMD

A multidisciplinary team approach cannot be over emphasized with thorough communication between the clinical team, infection prevention and control personnel, building infrastructure and engineering services, microbiologists and infectious diseases specialists, the hospital executive and hospital governance. Emphasis should be on (i) best practice antifungal treatments of patients with IFD; (ii) the safe placement of all patients, safe ventilation and water systems, and a safely built environment; and (iii) the review and remediation of cleaning procedures where relevant. In addition, surveillance procedures for IFD should be discussed.

A discussion of preferred, second-line and salvage therapies for individual mould infections and the rationale thereof is beyond the scope of the present review but is clearly detailed in a number of international guidelines for the management of IMDs [72,139,140,141], These guidelines address antifungal use and ancillary measures such as surgery and immunomodulation; as far as possible, they are based on evidence according to the Grading of Recommendations, Assessment, Development and Evaluation (GRADE) approach to assess and classify the supporting body of evidence [142,143]. Where evidence is lacking or is weak to support a recommendation, typically the recommendations are based on expert opinion.

Once a diagnosis is made, antifungal therapy should be instituted immediately or, if an IMD is suspected, instituted as soon as the treating clinician deems appropriate. The timing of antifungal therapy should occur once suspicion for an IMD is strong regardless of environmental studies, since many IMDs are medical emergencies.

### 7.1. Safe Patient Placement

Safe patient placement involves caring for patients in ward areas that are distant from any identified environmental contamination, including the use of protective environments such as HEPA-filtered rooms for those at high risk of IMDs (see above). If there is suspicion of a case cluster involving a compromised infrastructure, the transfering of the patients outside of this area should be undertaken immediately or as soon as practicable, and antifungal therapy should be carefully considered. Safe placement practice also requires eliminating obvious sources of mould within inpatient settings, such as potted plants and fresh or dried flowers [39].

The safe placement of patients is particularly challenging during periods of hospital construction or renovation, as these activities pose a period of increased risk where there are some reports observing that airborne conidia are dispersed during building work rendering airborne spread [28,98,130]. In this context, however, many experts are of the opinion that air primarily functions as the transmission vehicle rather than the primary source, although the source-transmission mode may be indistinct in situations such as defective HVAC systems [17]. A multidisciplinary approach is recommended to reduce the risk of IMDs during high-risk periods, including minimising dust dispersion and patient exposure and ensuring the regular cleaning and disinfection of surfaces [36].

Specifically, when the question of safe patient placement arises, attention is often drawn to air sampling to assist clinical decisions (see above and Table 2). However, of note, there are currently no international guidelines defining acceptable fungal loads nor recommendations for the frequency of air sampling across different clinical settings [85,86]. Nonetheless, the absence of opportunistic pathogenic moulds, such as *Aspergillus* species, in HEPA-filtered environments is generally considered desirable [19,85,86].

### 7.2. Cleaning Procedures

Once an outbreak has been identified, a walk-through of the affected hospital wards should be conducted to identify hazards by visual inspection, including fan settings, open windows, dirty air conditioners, duct contamination and inadequate filtration [144]. Following the identification of possible sites of infection control breaches, remediation is required. This includes enhanced cleaning: higher frequency cleaning using mould active disinfectants and the cleaning and disinfection of air conduits and circulation systems [36].

To prevent the spread of spores during construction, it is recommended that construction workers should not wear external clothing from the work area through clinical spaces [36]. If transit through a construction zone for patients is unavoidable, high-risk patients should be provided with N95/P2 respirator masks. Other infection prevention measures that can be considered during construction include dust trapping with sticky mats, zip wall hoarding, covering waste with plastic sheeting and water misting during demolition; however, there is a lack of evidence to support or refute these measures. The US Environmental Protection Agency does not recommend the routine use of fungicides, and their ineffectiveness has been supported by clinical studies [145,146]. Other cleaning procedures that may require remediation include hospital bed linen and laundry management. Expert opinion, including from the US CDC, suggests keeping healthcare textiles clean through washing processes that include disinfectants, air filtration and keeping surfaces free of lint [21,87].

### 7.3. Timeline of Surveillance

In a suspected outbreak, prospective active surveillance and retrospective case finding of probable and proven mould infection should occur and be instituted as soon as practicable (Figure 2). This is intuitive good sense, and the interrogation of both clinical records and laboratory information systems is recommended. If not already in place, a registry should be instituted to track cases of invasive mould infection [36]. Data collected should include the collection of post-mortem samples as well as serum galactomannan antigen and nucleic acid testing [6]. Surveillance should be targeted to high-risk patients and include measuring the breakthrough infection rate in high-risk patients on mould prophylaxis, mould-associated mortality and the background incidence of IMDs per bed occupancy day [147]. The surveillance of clinical cases in an outbreak investigation is suggested to occur for 12 months following the last positive case [6]. All of the above recommendations have not been tested against robust supportive evidence but represent pragmatic action steps in suspected IMD case clusters.

## 8. Conclusions

In conclusion, a solid understanding of mould ecology in healthcare settings, together with a clear identification of at-risk situations and patient populations, is essential for implementing specific preventive measures and diagnostic strategies that enable the timely diagnosis and management of healthcare-associated IMDs. Notably, atypical infections—whether in terms of pathogens, patient profiles, or unexpected incidence rates—should prompt early investigation, as they are more likely to reflect failures in infection control measures. Such investigations should aim to identify the source of infection to prevent further dissemination and include the search for additional cases as well as targeted environmental sampling. The high-resolution genotyping of isolates from different patients or from environmental sources can also be informative during suspected outbreaks; however, it is not sufficient on its own to rule out a mould outbreak in situations involving multiple introduction methods, such as defective ventilation systems. Finally, beyond targeted antifungal therapy and source control, the effective management of healthcare-associated IMDs requires a multidisciplinary team involving mycologists, infection prevention specialists, and infectious disease physicians.

## Figures and Tables

**Figure 1:**
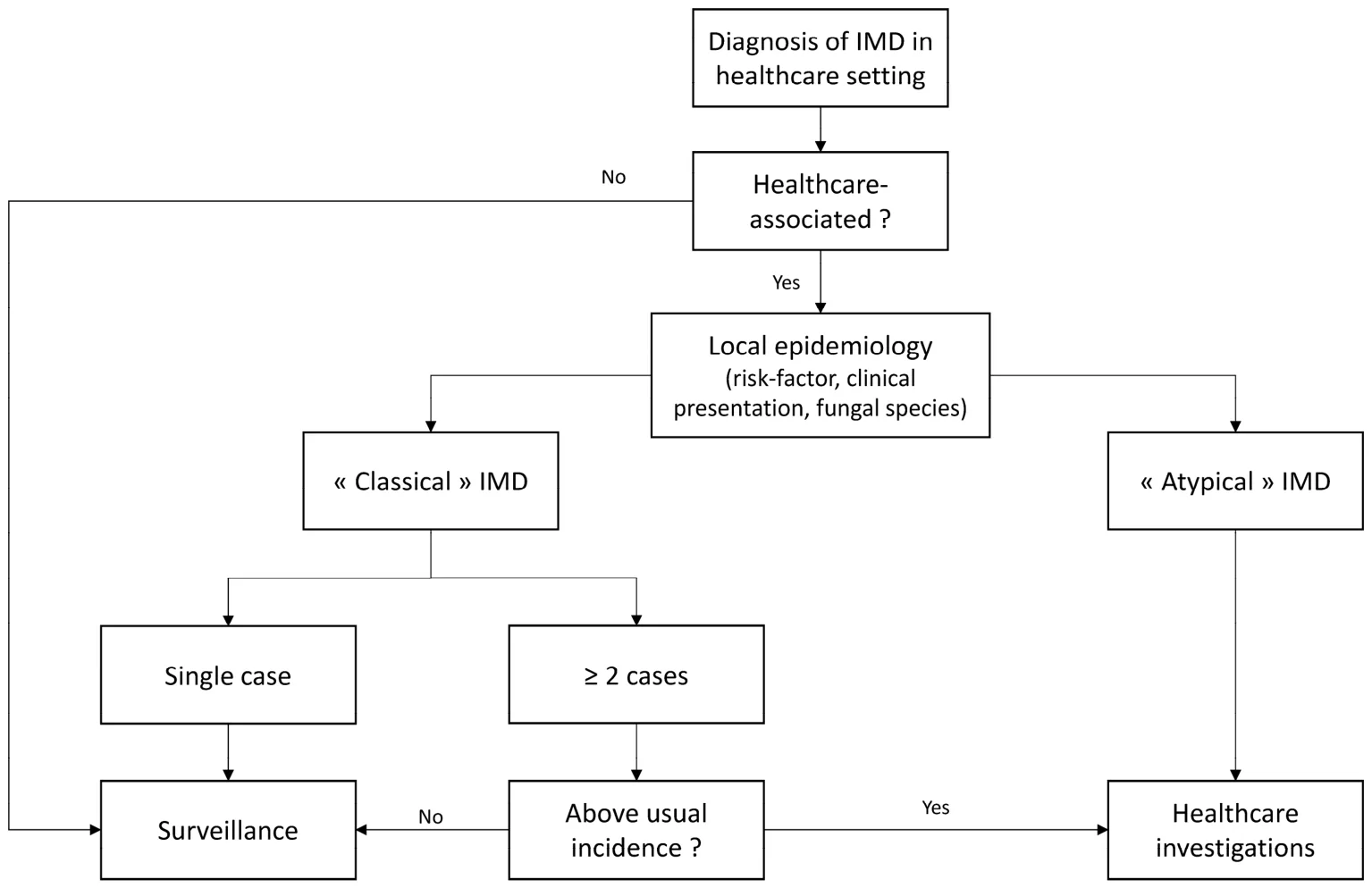
Conceptual framework for initiating healthcare investigations (IMD: Invasive mould disease). “Classical” and “Atypical” IMD should be classified according to the local epidemiology, including clinical presentation, patient’s risk factor and fungal species).

**Figure 2:**
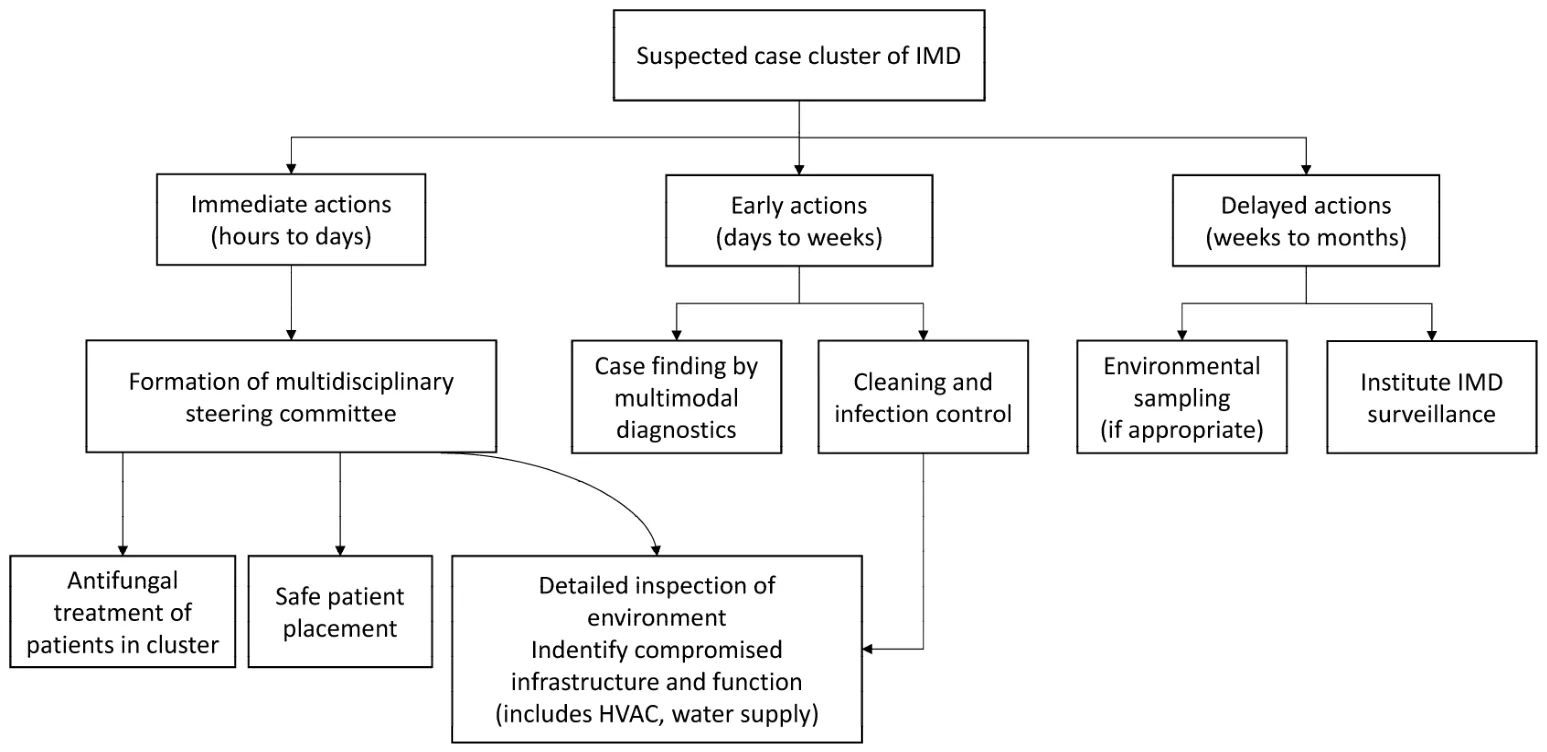
Practical approach to management of a suspected case cluster of invasive mould diseases with suggested timelines (HVAC: heating, ventilation and air conditioning; IMD: Invasive mould disease).

**Table 1:** Overview of clinical presentation, diagnostic strategies and interpretation by IMD risk group.

Risk Group		Usual Clinical Presentation	IMD Screening *	Targeted IMD Diagnosis *	Interpretation
**High-risk**	**Haematology patients**	Pulmonary and sinus infections Possible dissemination	Serum/plasma testing Neutropenic patients +++	Serum/plasma testing +/− Mycological examination of the suspected site of infection (if feasible)	Interpretation following EORTC/MSGERC criteria or other clinical guidelines Low sensitivity of serum testing non-neutropenic patients
	**Lung transplant patients**	Pulmonary infection	BAL testing	Mycological examination of the suspected site of infection +/− Serum/plasma testing	Interpretation following EORTC/MSGERC criteria or other clinical guidelines Possible lung colonisation by moulds Low sensitivity of serum testing non-neutropenic patients
	**Burn patients**	Invasive wound infections and skin necrosis Possible dissemination	Mycological examination of wound samples +/− Serum/plasma testing	Mycological examination of the suspected site of infection +/− Serum/plasma testing	Wound/necrotic tissues can be contaminated/colonized by environmental moulds
**Intermediate risk**	**Other organ transplant patients**	Pulmonary and sinus infections	No	Mycological examination of the suspected site of infection +/− Serum/plasma testing	Interpretation following EORTC/MSGERC criteria or other clinical guidelines
	**Intensive care patients**	Pulmonary infections	No	Mycological examination of the suspected site of infection +/− Serum/plasma testing	Interpretation following ICU guidelines
**Low risk**		Surgical or injection site infection Atypical presentation	No	Mycological examination of the suspected site of infection	Careful interpretation of positive results

* Serum/plasma testing includes fungal antigens and NAAT; BAL testing includes fungal culture, fungal antigens and NAAT; Mycological examination includes microscopic direct examination, fungal culture, NAAT and fungal antigens if available. Abbreviations: BAL: Broncho alveolar lavage; ICU: Intensive care unit; IMD: invasive mould disease; NAAT: nucleic acid amplification testing.

**Table 2:** Framework for environmental investigation of suspected healthcare-associated invasive mould disease according to clinical context.

Context	Air Sampling and Monitoring	Sampling of Water and Hard Surfaces
***Routine sampling and surveillance***		
Facility-wide	Unsupported application	Unsupported application
High-risk units e.g., haemopoietic stem cell transplant wards	Investigational application. Will vary with institution and region	Unsupported application
***Event-triggered scenarios***		
Suspected case cluster	Supported application	May be supported depending on context
HVAC failure assessment	Supported application	Not applicable
Water damage assessment	Supported application	Supported application
Engineering verification e.g., verifying high efficiency particulate air filtration, air pressures and air seal parameters	Supported application	Not applicable

Abbreviations: HVAC: heating, ventilation and air conditioning.

**Table 3:** *Aspergillus* healthcare-associated outbreaks where genotyping was performed.

Year Published, Country	Pathogen	Patient Type (No. Clinical Isolates)	Environmental Isolates (No.)	Outbreak Duration	Genotyping Method; Main Result	References
2002, USA	*Aspergillus fumigatus*	Transplant ICU (n = 8, 3 patients)	Air sample (n = 8)	1 month	RFLP; Identical genotypic profile for two patients and environmental isolates. Patient-to-patient transmission.	Pegues et al. [26]
2003, USA	*A. fumigatus* (n = 4) and *Aspergillus flavus* (n = 2)	Surgery unit (n = 6, 6 patients)	Settle plates (diverse moulds, n = 1 *A. fumigatus*) Duct surface samples (diverse *Aspergillus* species, n = 8 *A. fumigatus*, n = 3 *A. flavus*)	4 months	RAPD for *A. fumigatus*; Unrelated isolates Contamination from an air-handling unit in operating theatre.	Lutz et al. [112]
2011, UK	*A. fumigatus*	Premature neonates (n = 3, 3 patients)	Incubators and air samples (n = 17)	8 months	STR*Af*; Patients isolates closely related or undistinguishable to incubator isolates. No identical genotype across patients.	Etienne et al. [61]
2012, Spain	*A. fumigatus*, mixed species	Heart-surgery ICU (n = 6 *A. fumigatus*, 7 patients)	Air sample (n = 30)	17 months	STR*Af*; Matched genotype between patient and environmental isolates for three patients No identical genotype across patients.	Pelaez et al.; Guinea et al. [10,122]
2024, France	*Aspergillus flavus*	Premature neonates (n = 5, 4 patients)	Incubators and air samples (n = 10)	13 years	STR*Afla*, MALDI-ToF MS with convolutional neural network analysis; Matched genotype between patient and incubator isolates for two patients. One patient had a different genotype from the incubator. No identical genotype across patients.	Mohammad et al. [20]
2024, The Netherlands	*A. fumigatus* (n = 8) and *A. flavus* (n = 3)	ICU (n = 11, 7 patients)	Air sample (n = 4 *A. fumigatus*)	10 days	STR*Af*, WGS; Different STR*Af* profiles across environmental and clinical isolates. Mean of 19,055 SNPs by WGS between *A. fumigatus* isolates. Contamination via construction work.	Hiel et al. [121]
2025, France	Azole resistant *A. fumigatus*	Interventional bronchoscopy service (n = 9, 9 patients)	Surface swabs (n = 4)	12 months	STR*Af*, WGS; Same STR*Af* genotype for eight of the nine isolates from the outbreak. Median of 1297 SNPs [IQR 759–1421] by WGS. Unrelated environmental isolates.	Joste et al. [110]
2026, Netherlands	*A. flavus*	Haematology (n = 35, 21 patients)	Air and surface samples (n = 110)	6 years	STR*Afla*, WGS; 28 unique STR*Afla* genotypes with one dominant genotype shared by 11 patients and 82 environmental samples. Outbreak isolates clustered together by WGS SNPs analysis. Introduction of the outbreak strain from building material.	Gewecke et al. [100]

Abbreviations: MALDI-ToF MS: matrix-assisted laser desorption time-of-flight mass spectroscopy; RAPD: random amplification of polymorphic DNA; RFLP: restriction fragment length polymorphism; SNPs: single nucleotide polymorphisms; STR*Af*: short tandem repeat for *A. fumigatus*; STR*Afla*: short tandem repeat for *A. flavus*; WGS, whole-genome sequencing.

**Table 4:** Outbreaks due to non-*Aspergillus* filamentous fungi: major healthcare-associated clusters where genotyping was performed (updated from Douglas et al. [130]).

Year Published, Country	Pathogen	Patient Type (No. Clinical Isolates)	Environmental Isolates (No.)	Outbreak Duration	Genotyping Method; Main Result	References
**Mucorales**						
2000, 2019 Germany	*Cunninghamella bertholletiae*	Haematology, renal transplantation (n = 4, 4 patients)	None	2 years	Rep-PCR. RAPD, STR; All strains clonal.	Verhasselt et al.; Rickerts et al. [113,131]
2018, France	*Mucor circinelloides*	Burn, (n = 14, 6 patients)	None	2 years	WGS; SNP distance among outbreak isolates ranging from 20,000 to >200,000. Unrelated isolates.	Garcia-Hermoso et al. [128]
2018, Argentina	*Rhizopus microsporus*	Post knee construction surgery, (n = 3, 3 patients)	None	7 months	RAPD; Two patients with the same genotype. Third patients clustered together but not identical.	Gamarra et al. [114]
2019, Canada	*Rhizomucor pusillus* (n = 2) and *Lichtheimia corymbifera* (n = 1)	Heart-lung transplant (n = 3, 3 patients)	None	6 months	WGS; SNP distance among the two *R. pusillus* isolates > 5900 SNPs Unrelated isolates.	Marek et al. [129]
2020, USA	*R. microsporus* (n = 2), *Rhizopus arrhizus* (n = 1), *L. corymbifera* (n = 1)	Solid organ transplantation (n = 5, 4 patients)	Linen-associated (n = 45)	10 months	WGS; The two *R. microsporus* outbreak isolates highly similar by core phylogeny and global genome features. No link with environmental isolates.	Nguyen et al. [120]
***Scedosporium* and *Lomentospora* species**						
1997, Spain	*Lomentospora prolificans*	Hematology/oncology (n = 14, 4 patients)	None	1 month	RAPD; Two patients with same genotype. Remaining patients infected by two different genotypes.	Ruiz-Diez et al. [115]
2002, Spain	*L. prolificans*	Hematology/oncology (n = 6, 6 patients)	Air sample (n = 2)	15 months	RAPD; Identical genotype across the six patients and environmental samples.	Guerrero et al. [116]
2015, Germany	*Scedosporium boydii*	Post-surgical (n = 5, 5 patients)	None	14 months	MLST; Identical genotype across the five patients.	Bernhardt et al. [132]
***Fusarium* species**						
2014, USA	*Fusarium incarnatum-equiseti* species complex	Vitrectomy surgery; *F. incarnatum-equiseti* species complex (7, 7 patients)	Brilliant Blue G vial (n = 1)	19 months	MLST; Identical genotype for four clinical and one environmental *Fusarium* isolates.	Mikosz et al. [133]
2017, Brazil	*Fusarium oxysporum* species complex	Paediatric cancer (n = 7, 7 patients)	Catheter tip (n = 7)	5 months	AFLP; All clinical and catheter isolates genetically similar.	Carlesse et al. [134]
2022, South Korea	*F. oxysporum* species complex	Post cataract surgery (n = 39, 50 patients)	Ocular viscoelastic device (n = 13)	4 months	MLST; 12 clinical and two environmental strains shared the same genotype.	Yoon et al. [60]
2024, Mexico	*Fusarium solani* species complex (80 patients)	Neuraxial blockade (epidural)	None	11 months	Not performed (see below).	Garcia-Rodriguez et al. [65]
2026, Mexico *	*F. solani* species complex (24, 24 patients)	Post epidural anaesthesia	None	5 months	WGS (n = 19 isolates); Two isolates from Matamoros outbreak, one from Durango, 16 unrelated strains, two Matamoros strains separated by 104 SNPs.	Smith et al. [64]
**Other species**						
2013, 2014, USA	*Exserohilum rostratum*	Post methylprednisolone injection (n = 22, 750 patients)	Methylprednisolone vials (n = 6)	9 months	WGS; SNP distance among outbreak and vials isolates ≤ 8 SNPs confirming the common source of infection.	Chiller et al.; Litvinsetva et al. [67,135]
2016, Chile, Columbia	*Sarocladium kiliense*	Oncology (n = 18, 67 patients)	Ondansetron vials (n = 7)	6 months	WGS; SNP distance among outbreak and vials isolates < 5 SNPs	Etienne et al. [136]

* Comprises analysis of both the Matamoros and Durango outbreaks [64,65]. Abbreviations: AFLP, amplified fragment length polymorphism; MLST, multilocus sequence typing; RAPD, random amplification of polymorphic DNA; SNPs, single-nucleotide polymorphisms; WGS, whole-genome sequencing.

## Data Availability

No new data were created or analyzed in this study. Data sharing is not applicable to this article.
